# Supplementary material for: Venomix: a simple bioinformatic pipeline for identifying and characterizing toxin gene candidates from transcriptomic data
Source: PeerJ. 2018 Jul 31;6:e5361. doi: 10.7717/peerj.5361 (PMC6074769; doi:10.7717/peerj.5361)
Supplement: Supplemental Information 3 [file peerj-06-5361-s003.gz › FinalOutput_E-20/Astacin-like_metalloprotease_toxin_5_1/finaltree.pdf]

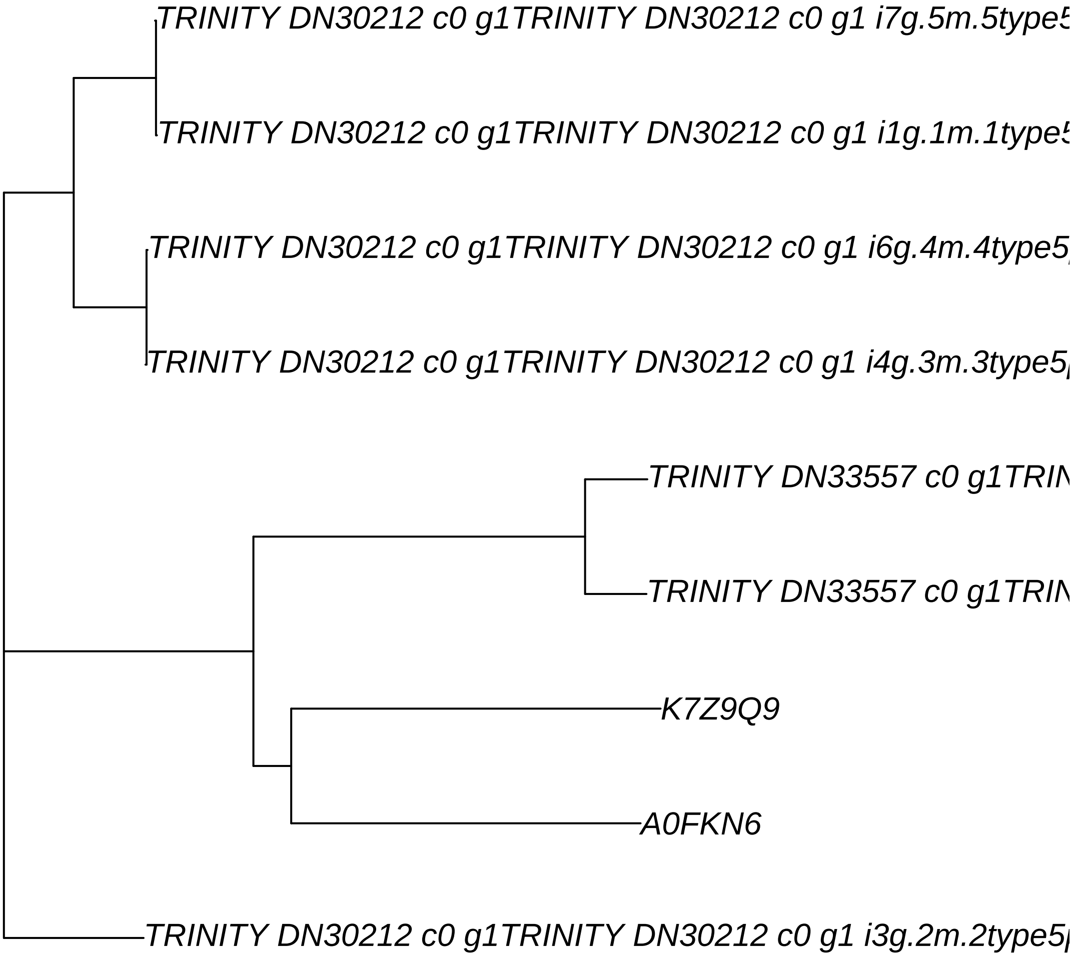

TRINITY DN30212 c0 g1TRINITY DN30212 c0 g1 i7g.5m.5type5
TRINITY DN30212 c0 g1TRINITY DN30212 c0 g1 i1g.1m.1type5
TRINITY DN30212 c0 g1TRINITY DN30212 c0 g1 i6g.4m.4type5
TRINITY DN30212 c0 g1TRINITY DN30212 c0 g1 i4g.3m.3type5
TRINITY DN33557 c0 g1TRIN
TRINITY DN33557 c0 g1TRIN
K7Z9Q9
A0FKN6
TRINITY DN30212 c0 g1TRINITY DN30212 c0 g1 i3g.2m.2type5
